# Coordination and Crosstalk between Autophagosome and Multivesicular Body Pathways in Plant Stress Responses

**DOI:** 10.3390/cells9010119

**Published:** 2020-01-03

**Authors:** Mengxue Wang, Xifeng Li, Shuwei Luo, Baofang Fan, Cheng Zhu, Zhixiang Chen

**Affiliations:** 1 College of Life Sciences, China Jiliang University, 258 Xueyuan Street, Hangzhou 310018, Zhejiang, China; 15516515057@163.com (M.W.); 19a0902115@cjlu.edu.cn (X.L.); luoshuwei@163.com (S.L.); 2 Department of Botany and Plant Pathology and Purdue Center for Plant Biology, Purdue University, 915 W. State Street, West Lafayette, IN 47907-2054, USA; bfan@purdue.edu

**Keywords:** autophagy, multivesicular body (MVB), plant stress response, protein degradation, vesicle trafficking

## Abstract

In eukaryotic cells, autophagosomes and multivesicular bodies (MVBs) are two closely related partners in the lysosomal/vacuolar protein degradation system. Autophagosomes are double membrane-bound organelles that transport cytoplasmic components, including proteins and organelles for autophagic degradation in the lysosomes/vacuoles. MVBs are single-membrane organelles in the endocytic pathway that contain intraluminal vesicles whose content is either degraded in the lysosomes/vacuoles or recycled to the cell surface. In plants, both autophagosome and MVB pathways play important roles in plant responses to biotic and abiotic stresses. More recent studies have revealed that autophagosomes and MVBs also act together in plant stress responses in a variety of processes, including deployment of defense-related molecules, regulation of cell death, trafficking and degradation of membrane and soluble constituents, and modulation of plant hormone metabolism and signaling. In this review, we discuss these recent findings on the coordination and crosstalk between autophagosome and MVB pathways that contribute to the complex network of plant stress responses.

## 1. Introduction

The trafficking, degradation, and recycling of cellular components are essential for the growth and survival of all cellular organisms. Autophagy is an important pathway for the degradation of intracellular components, including organelles, proteins, and RNAs, in eukaryotic cells [1,2,3]. Under normal growth conditions, autophagy is suppressed and operates only at a low basal level. Upon exposure to stress conditions, such as nutrient starvation, autophagy is induced with the formation of a cup-shaped structure called a phagophore or isolation membrane, which can expand to engulf cytoplasmic components to form a closed double-membraned structure termed autophagosomes [4]. The autophagosomes fuse with the lysosomes/vacuoles to degrade the sequestered cargo. Multivesicular bodies (MVBs) are specialized endosomes in the endocytic pathway that function in the internalization, transport, sorting, and degradation of specific plasma membrane proteins, such as membrane receptors and ion transporters [5]. MVBs contain intraluminal vesicles generated from invagination and budding of the limiting membrane through the action of protein complexes named ESCRT-0, I, II, and III (endosomal sorting complex required for transport) [5]. The content of MVBs can be degraded through fusion with lysosomes/vacuoles or recycled into the cell surface after fusion with the plasma membrane. The endocytic and MVB pathway has a cellular housekeeping role and operates even under normal growth conditions but is subjected to tight regulation in response to developmental and environmental cues to ensure the correct delivery of membrane cargo [6,7].

Both autophagy and MVBs play a critical role in plant responses to a broad spectrum of biotic and abiotic stress conditions [1,8,9,10]. Both the formation of the autophagosomes and expression of autophagy-related genes (*ATG*s) are induced under nutrient starvation, biotic and abiotic stresses, such as oxidative, high salt, and osmotic stress conditions [1,11,12,13]. *Arabidopsis* autophagy mutants are hypersensitive to these abiotic stresses [8,12,14,15,16,17,18,19]. Autophagy also affects plant responses to microbial pathogens [13]. Depending on the age of plants and the stage of infection, autophagy can either promote or suppress defense-associated hypersensitive cell death against biotrophic pathogens [13,20,21,22,23]. Autophagy plays a critical role in plant resistance to necrotrophic fungal pathogens [11,20] and in plant–virus interactions [24,25]. Likewise, the endocytic and associated MVB pathways are essential for the regulation of the levels and activity of plasma membrane proteins in response to changing environmental conditions. The presence of excess levels of metal ions in the soil can trigger downregulation of their corresponding ion transporters in the plasma membrane through increased endocytosis [26,27]. Over the past decade or so, important progress has also been made in establishing the critical roles of MVBs and associated trafficking in pathogen recognition, defense signaling, and deployment of defense-related molecules during plant responses to microbial pathogens [6,9]. Both the endocytic activity and MVB biogenesis are induced in plant cells upon biotic and abiotic stresses [9,10]. In *Arabidopsis*, pathogen- and stress-induced endocytosis and MVB biogenesis are abolished in the mutants for LIP5 (lyst-interacting protein 5) [9,10]. LIP5 is an activator of the SKD1 (suppressor of K^+^ transport growth defect 1) ATPase, a core component in the ESCRT pathway central to MVB biogenesis [9,10]. Importantly, the *lip5* mutants are compromised not only in plant disease resistance but also in plant tolerance to salt and heat stresses [9,10]. These results confirm a critical role of stress-induced, LIP5-dependent MVB biogenesis in plant responses to both biotic and abiotic stresses.

As two closely related partners in the lysosomal/vacuolar protein degradation system, there are extensive interactions in both the regulation and functions between the autophagosome and MVB pathways based on studies in yeast and animals [28,29,30,31]. For example, the maturation process of autophagosomes in some organisms often includes fusion events with early and late endosomal vesicles, including MVBs, to form a hybrid organelle called the amphisome prior to the fusion with lysosomes (forming an autolysosome) to degrade the incorporated materials [29]. The MVB pathway and autophagy are also coordinated in action to ensure cell survival during starvation in yeast cells [30]. In plants, extensive studies have uncovered not only conserved and unique components but also shared factors in the biogenesis and trafficking of autophagosomes and MVBs, which have been discussed in recent reviews [32,33]. With well-established common roles in plant stress responses, how the two related protein trafficking pathways act together to protect plants under environmental stresses is very important and has gained increasing interest in recent years. Progress has been made in the analysis of the interplay of signaling pathways that regulate stress-induced biogenesis of autophagosomes and MVBs [9,34]. More importantly, recent studies have provided strong evidence for the coordinated action of the autophagosome and MVB pathways during plant responses to biotic and abiotic stresses. In this review, we discuss these recent progresses in the analysis of the crosstalk and coordination between these two related trafficking pathways in plant stress responses.

## 2. Stress Regulation of Autophagosome and MVB Biogenesis

Autophagy occurs at a low basal level in plant cells under normal growth conditions but can be induced by biotic and abiotic stresses, such as nutrient starvation [35,36]. Upon nutrient, osmotic, or salt stress in plant cells, the Snf1 (sucrose non-fermenting 1)-related protein kinase 1 (SnRK1), a master regulator of metabolism, can activate autophagy by inhibiting the TOR (target of rapamycin) complex, a negative regulator of autophagy [4]. Inhibited TOR leads to activation of the ATG1 complex or the deactivation of the ribosomal p70 S6 kinase (S6K) and type 2A-phosphatase-associated protein 46 kD (Tap46), leading to autophagy activation [4]. Autophagy activation by oxidative and endoplasmic reticulum (ER) stress, however, is mediated by the TOR-independent pathways through direct activation of the ATG1 complex by SnRK1 [4]. Autophagy activation by ER stress can also be triggered by accumulated unfolded proteins through the activation of the IRE1b (inositol-requiring enzyme 1b) serine/threonine-protein kinase/endoribonuclease, a master regulator of ER stress responses [4,15]. After autophagy activation, autophagosome formation in plant cells follows a conserved process involving phagophore initiation, autophagosome expansion, maturation, and degradation. It has been shown that a number of ESCRT- and MVB-associated proteins play important roles in the process of autophagosome formation in plants [32,33]. For example, *Arabidopsis* SH3P2 (SH3 domain-containing protein 2), a ubiquitin-binding protein that acts with ESCRT-I and the deubiquitylating enzyme AMSH3 (associated molecule with the SH3 domain of STAM3) in the transfer of ubiquitinated proteins to the ESCRT machinery [37], also binds phosphatidylinositol 3-phosphate and ATG8 and regulates autophagosome formation in *Arabidopsis* [38,39]. Analysis using RNAi (RNA interference) and other approaches suggests that SH3P2 is a key regulator of plant autophagy most likely by facilitating membrane expansion or maturation during autophagosome formation [38,39]. FREE1 (FYVE domain protein required for endosomal sorting 1), another *Arabidopsis* protein that binds to phosphatidylinositol-3-phosphate and ubiquitin, is associated with MVBs through interaction with the ESCRT-I subunit VPS23 (vacuolar protein sorting 23) and ESCRT-III subunit Snf7 (sucrose non-fermenting protein 7) [40]. FREE1 also interacts with SH3P2 on autophagosomes [40]. The mutation of FREE1 leads to the formation of abnormal MVB-autophagosome hybrid structures, further supporting the interactions of the two related types of vesicles [40].

The endocytic and associated MVB pathway operates at high levels even under normal growth conditions and is essential for plant growth and development [41,42]. However, both the endocytic activity and MVB biogenesis are regulated in plant cells in responses to changes in environmental cues, particularly to stress conditions. Upon pathogen infection, plasma membrane-localized pattern recognition receptors, including the receptor-like kinases FLS2 (flagellin-sensitive 2) and CERK1 (chitin elicitor receptor kinase 1), are ubiquitinated and internalized through endocytosis for recycling or degradation upon binding to pathogen elicitors to modulate plant immune responses [43,44]. High levels of ions in soil can cause increased turnover through endocytosis of various plasma membrane-localized ion channels, including the boric transporter BOR1 (require high boron1) [45], the iron transporter IRT1 (iron-regulated transporter 1) [26,27], the phosphate transporters PHTs [46], and the nitrate transporter NRT1.7 [47]. Likewise, increased biogenesis of MVBs has been observed in plant cells upon infection by microbial pathogens or exposure to abiotic stress, such as high salt [9,10]. Therefore, there are constitutive MVB biogenesis and trafficking required for normal growth and development as well as induced MVB biogenesis and trafficking in response to altered environmental conditions. This notion of constitutive and induced MVB biogenesis is supported by the recent characterization of *Arabidopsis* LIP5 protein, a positive activator of the SKD1 AAAA ATPase, which catalyzes the disassembly of the ESCRT III complex during MVB biogenesis [9,10]. Unlike mutants for other ESCRT components, such as SKD ATPase, which are often lethal [48], knockout mutants for LIP5 grow and develop normally, indicating that the basal SKD ATPase activity is sufficient for the constitutive levels of MVB biogenesis required for plant growth and development [9,10]. However, the *lip5* mutants are highly susceptible to microbial pathogens and severely compromised in tolerance to salt and heat stress [9,10]. The compromised disease resistance and stress tolerance of the *lip5* mutants are associated with defects in pathogen- and stress-induced endocytosis and MVB biogenesis [9,10]. These results indicate that LIP5 is a key regulator of stress-induced MVB biogenesis in plant responses to both biotic and abiotic stresses.

**Figure 1:**
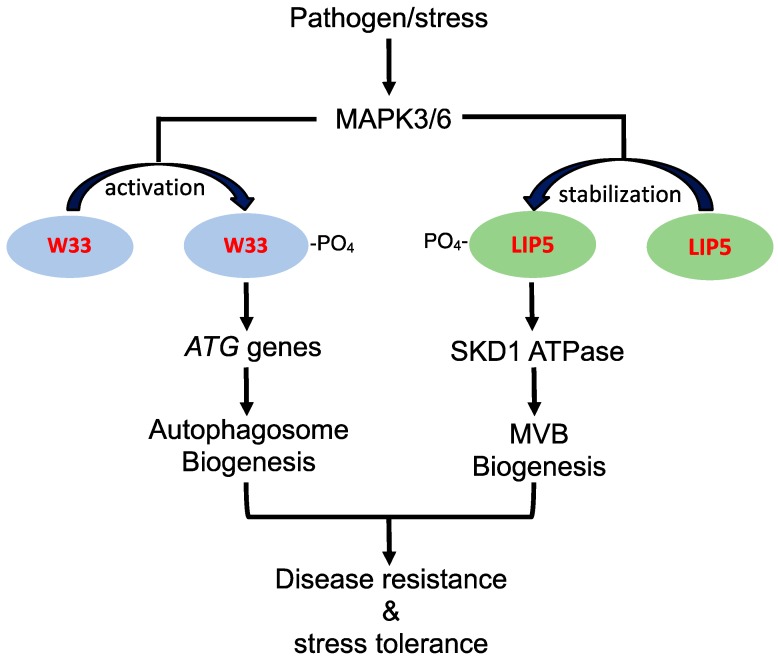
Role of pathogen/stress-responsive MAPK3/6 signaling cascade in stress-induced autophagosome and MVB biogenesis. Pathogen/stress-activated MAPK3 and 6 phosphorylate and activate WRKY33 (W33) transcription factor to activates ATG gene transcription for sustained autophagy induction. Pathogen/stress-activated MAPK3 and 6 also phosphorylate LIP5 to improve its stability for stimulation of SKD1 required for stressed-induced MVB biogenesis. SDK1 is an AAA ATPase that catalyzes the release of the ESCRT-III complex from the membrane during the biogenesis of MVBs. Induced autophagosome and MVB biogenesis promote plant disease resistance and stress tolerance.

There are also studies supporting that the regulatory pathways for stress-induced autophagy and MVB pathways intersects in the complex network of plant stress responses. In *Arabidopsis*, both autophagy and WRKY33 transcription factor, which belongs to a family of DNA-binding proteins containing a conserved WRKYGQK motif, play an important role in plant resistance to necrotrophic pathogens [49,50]. WRKY33 interacts with autophagy-related protein ATG18a and is required for sustained induction of *ATG18a* gene expression by pathogen infection [11]. Furthermore, sustained induction autophagosome formation by pathogen infection is compromised in the *wrky33* mutants [11]. These results indicate that the critical role of WRKY33 in plant immune response is in part mediated through its positive regulation of pathogen-induced autophagy. WRKY33 is a target of stress- and pathogen-responsive MAPK3 and 6 (mitogen-activated protein kinase 3 and 6), which phosphorylate and activate the transcription factor [34] (Figure 1). Interestingly, the LIP5 positive regulator of stress-induced MVB biogenesis is also an interacting protein and target of MAPK3 and 6 (Figure 1). Under normal growth conditions, unphosphorylated LIP5 is unstable and subjected to degradation [9,10]. Upon pathogen infection or salt treatment, activated MAPK3 and 6 phosphorylate LIP5 and increase its stability [9,10]. As a result, LIP5 protein levels increase in plant cells upon exposure to stress conditions, leading to stimulation of SKD1 ATPase and induced MVB biogenesis (Figure 1). Therefore, the stress-responsive MAPK3/6 signaling cascade, which plays a central role in plant growth, development, and stress responses [51], appears to regulate pathogen- and stress-induced formation of both autophagosomes and MVBs, supporting coordinated regulation of the two related trafficking pathways during plant stress responses (Figure 1).

## 3. Autophagy and MVB Coordination in Plant Biotic Stress Response

**Figure 2:**
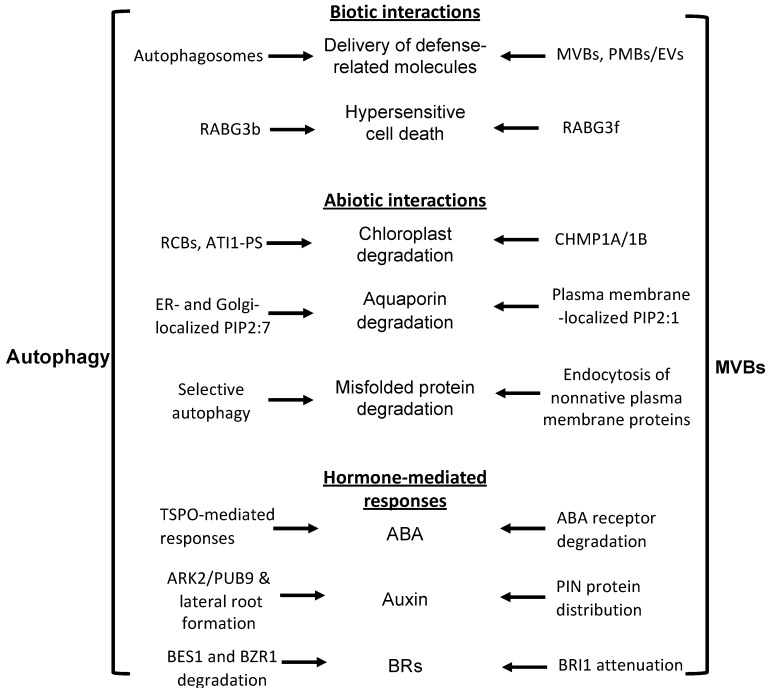
Coordination and crosstalk between the autophagosome and MVB pathways in plant biotic and abiotic interactions and in plant hormone-regulated stress responses.

Both autophagy and MVB pathways play important and complex roles in plant immune responses to microbial pathogens [6,13]. One of the critical roles of vesicle trafficking in the plant immune system is the mobilization and trafficking of defense-related molecules to the plant cell surface against invading pathogens. MVBs plays a critical role in the cell surface defense in plant cells. When plants are infected by filamentous pathogens, the germinating spores of the pathogens develop infection pegs on the leaf surface to invade the epidermal cells, which can induce plant defense responses, including the formation of local cell wall appositions (papilla) at the attack sites [52]. MVBs and cell wall-associated paramural bodies (PMBs) accumulate in the vicinity of pathogen-induced papillae [53,54,55]. PMBs are situated between the cell wall and the plasma membrane, likely resulting from the fusion of MVBs with the plasma membrane [55]. The accumulation of MVBs and PMBs occurs near papillae in plant cells infected not only by pathogenic fungal pathogens, but also by bacteria, and nematodes for the delivery of defense-related molecules, including phytoalexins, callose, and reactive oxygen species (ROS), to papillae [56,57,58]. The fusion of MVBs with the plasma membrane can also lead to the generation of plant extracellular vesicles (EVs). EVs from *Arabidopsis* leaves contain proteins involved in the metabolism and transport of defense-related molecules, including small RNAs, and their secretion is enhanced in *Arabidopsis* after *Pseudomonas syringae* infection [59,60,61]. EV protein profiles are most similar to those from the TGN(*trans* Golgi network)/MVB compartments, supporting that EVs are derived from the intralumenal vesicles of MVBs [60,61]. We have also shown that in *Arabidopsis*, both the endocytosis and MVB biogenesis were induced in an LIP5-dependent manner after infection by the bacterial pathogen *P. syringae* [9]. Pathogen infection resulted in increased numbers of MVBs and PMBs in wild-type plants but not in the *lip5* mutants [9]. These results provide genetic evidence for a critical role of pathogen-induced, LIP5-dependent MVB biogenesis in the formation of defense-related vesicles at the infection sites (Figure 2).

Upon successful penetration, filamentous pathogens can develop haustoria, a special feeding structure, into plant cells. Each haustorium is surrounded by the plasma membrane of the plant cell termed extrahaustorial membrane (EHM), which is likely synthesized de novo [62]. In *Arabidopsis*, Rab5 GTPase is an MVB marker that accumulates in the EHM after infection with a powdery mildew fungus [63]. Cell surface immune receptors, such as FLS2 and RPW8 (resistance to powdery mildew 8) resistance proteins, are also recruited to the EHM upon pathogen infection, likely as a host border control mechanism at the plant–pathogen interface [62]. In tobacco (*Nicotiana benthamiana*) cells invaded by the oomycete pathogen *Phytophthora infestans*, RabG3c, an Rab7 GTPase and an MVB marker, but not a tonoplast-localized sucrose transporter, is also recruited to the EHM [64]. Specific rerouting of MVBs from the vacuole to the host–pathogen interface may participate in the formation of the EHM and surface defense. Interestingly, recent studies have revealed that perihaustorial compartments are also the hotspots for autophagosome biogenesis. Defense-related autophagosomes labeled by the ATG8CL and NBR1(neighbor of BRCA1 gene 1) autophagy markers are diverted to the EHM in tobacco cells upon infection of *P. infestans* [65]. Overexpression of both ATG9 and NBR1 led to increased resistance while silencing of NBR1 caused increased disease lesions [65,66]. These results indicate that increased autophagy activity at the plant–pathogen interface contributes to plant defense. The roles of pathogen-induced NBR1-mediated selective autophagy in the plant interaction with the pathogen are further supported by the finding that the PexRD54 effector of *P. infestans* binds host autophagy protein ATG8CL to deplete the autophagy cargo receptor NBR1 out of ATG8CL complexes, thereby antagonizing NBR1′s positive effect on pathogen defense [66]. Therefore, both defense-related autophagosomes and MVBs are mobilized to the plant–pathogen interface to restrict pathogens during plant interactions with filamentous pathogens. These observations support the coordinated roles of autophagy and MVB pathways in plant surface defense against invading pathogens (Figure 2).

Pathogen-induced host hypersensitive cell death is a critical process that can often determine the outcomes of plant interactions with microbial pathogens [67]. Both the autophagosome and MVB pathways participate in the regulation of plant hypersensitive cell death upon pathogen infection. Studies on plants Rab GTPases, regulators of vesicle trafficking, also indicate crosstalk between autophagosome and MVB pathways in immunity-associated hypersensitive cell death. In endocytosis in yeast and animals, proteins endocytosed from the plasma membrane pass through the early endosomes to the MVB late endosomes before the fusion with the lysosomes/vacuoles. The maturation of the early endosomes to MVBs involves Rab5 to Rab7 GTPase conversion. This conversion requires Mon1 (monensin sensitivity 1) protein, which acts together with CCZ1 (calcium caffeine zinc sensitivity 1) as a guanine-nucleotide exchange factor for Rab7 activation and Rab5 inactivation [68]. In *Arabidopsis*, there are eight genes encoding putative Rab7 proteins [68]. Genetic analysis with different combinations of *rab7* mutants indicate that their functions are highly redundant [68]. Analysis of RABG3f, a highly expressed member of the *Arabidopsis* Rab7 family, showed that it is localized to MVBs and the vacuoles [68]. Expression of a dominant-negative form of the Rab7 protein in *Arabidopsis* led to the formation of enlarged MVBs, altered vacuole morphology, inhibited vacuolar trafficking, blocked degradation of storage proteins in the protein storage vacuole, and caused seedling death [68]. These results indicate that Rab7 proteins are required for both MVB-to-vacuole trafficking and vacuole biogenesis. On the other hand, analysis of another Rab7 protein family member, RabG3b, indicates a positive role in autophagy and hypersensitive cell death in response to pathogen infection [69,70]. Expression of a constitutively active RabG3b (RabG3bCA) in transgenic *Arabidopsis* plants led to accelerated, unrestricted programmed cell death within one day of infection by avirulent strains of the bacterial pathogen *P. syringae* [69,70]. By contrast, the autophagy-defective *atg5-1* mutant gradually developed chlorotic cell death through uninfected sites over several days [69,70]. Microscopic analyses showed the accumulation of autophagic structures during hypersensitive cell death in RabG3bCA cells [69,70]. These results suggest that RabG3b contributes to hypersensitive cell death through the activation of autophagy, which plays a positive role in plant immunity-triggered programmed cell death during the hypersensitive response. Thus, members of the Rab7 protein family regulate both MVB and autophagosome pathways during plant responses to pathogen infection (Figure 2).

## 4. Autophagy and MVB Coordination in Plant Abiotic Stress Response

Autophagy plays a critical role in nutrient recycling during leaf senescence or under nutrient starvation [1,71]. Due to the abundance in proteins and other molecules, chloroplasts are dismantled during leaf senescence or under N or C starvation and their constituents are transported by autophagosomes to vacuoles for degradation through several pathways [72]. Chloroplast stromal proteins, including Rubisco, are transported into the small double membrane structures called Rubisco-containing bodies (RCBs). The autophagic nature of RCBs is supported by the colocalization of RCBs labeled by a chloroplast-targeted red fluorescent protein with the GFP-ATG8 autophagosome marker [73]. Delivery of small starch granules (SSGs) from chloroplasts to vacuoles is carried out by plastid-derived small spherical structures called SSG-like bodies in an autophagy-dependent manner [74]. The selective autophagy receptor ATI1 (ATG8-interacting Protein 1) also mediates the delivery of chloroplast components to vacuoles for degradation [75]. ATI1-plastid associated bodies (ATI-PS), which contain thylakoid membrane proteins and chlorophylls, are detected in the periphery and inside of plastids [75]. ATI1-PS bodies are released from chloroplasts into the cytosol independent of the autophagic machinery [75]. However, their fusion with the central vacuole requires functional autophagy [75]. In addition, when cells are subjected to UV-induced damage, entire chloroplasts can be engulfed by autophagosomal structures [76].

The ESCRT-III subunit paralogs CHMP1A (charged MVB protein1) and CHMP1B play a direct role in the autophagic degradation of plastid proteins in *Arabidopsis* [77]. Specifically, the two homologs of the ESCRT-III component are required for the transport of RCB cargo into the vacuoles [77]. Like autophagy mutants, *chmp1* mutant plants hyperaccumulated plastid clusters with plastid proteins, including proteins involved in plastid division [77]. Autophagy was increased in *chmp1* based on an increase in vacuolar GFP cleavage from the autophagic reporter GFP-ATG8. However, autophagic degradation of the stromal cargo RECA-GFP was greatly reduced in the *chmp1* plants upon starvation [77]. Thus, it appears that the autophagy machinery is responsible for the release of RCB bodies containing plastid material into the cytoplasm, whereas CHMP1 proteins are required for the delivery of RCB bodies to the vacuole. Therefore, ESCRT components and the autophagy machinery act in coordination in the delivery of chloroplast proteins to the vacuoles for degradation and nutrient recycling under starvation (Figure 2).

Coordination and crosstalk between endocytic MVB and autophagosome pathways are also involved in plant responses to water-related abiotic stresses caused by salt, osmotic, and drought conditions. Water flow across plant cell membranes is modulated by aquaporins in the plasma membrane and the tonoplast to regulate growth and transpiration [78]. The protein levels and activities of aquaporins are constantly regulated at the levels of transcription, protein stability, subcellular trafficking, and gating [78]. Generally speaking, the expression of most aquaporin genes is downregulated upon exposure to water-related stress, such as drought or salt stress conditions, probably to decrease the water permeability of the root, thereby limiting water loss and potentially creating a hydraulic signal for the induction of stomatal closure [78]. Recent studies have shown that both MVB and autophagosome pathways are involved in the degradation of plasma membrane-localized aquaporin proteins in response to abiotic stress responses. In *Arabidopsis*, salt stress reduces the fluorescence of GFP(green fluorescence protein)-labeled aquaporin PIP2;1 (plasma membrane intrinsic protein 2;1) at the plasma membrane and increased it in the vacuolar lumen [79]. The internalization of PIP2;1 is inhibited by inhibitors of clathrin-mediated endocytosis and phosphatidylinositol 3-kinase (PI3K) and phosphatidylinositol 4-kinase (PI4K) associated with vesicle trafficking [79]. Inhibiting PI4K and PI3K suppresses salt-induced endocytosis at the plasma membrane and trafficking after internalization of GFP-PIP2;1 [79]. These results suggest that salt stress induces the internalization of PIP2;1 from the plasma membrane through endocytosis to the vacuolar lumen though MVBs in a manner that is dependent on clathrin, PI3K, and PI4K.

Interestingly, a recent study has shown that another plasma membrane aquaporin, PIP2;7, is downregulated by the multi-stress regulator TSPO (outer membrane tryptophan-rich sensory protein) through a selective autophagic pathway during plant stress responses [80]. In *Arabidopsis*, the polytopic and stress-responsive membrane protein TSPO is localized in the Golgi apparatus, where its levels are tightly regulated by various mechanisms, including selective autophagy [80]. TSPO interacts with the plasma membrane aquaporin PIP2;7 at the ER and Golgi membranes in planta [80]. Overexpression of TSPO reduced the accumulation of overexpressed PIP2;7 in the plasma membrane and abolished the membrane water permeability mediated by transgenic PIP2;7 [80]. Inhibition of autophagy increased the stability of both TSPO and PIP2;7, suggesting that the autophagic pathway is responsible for the degradation of the complex containing both TSPO and PIP2;7 [80]. These results support a critical role for TSPO through a selective autophagy pathway in regulating the protein levels of PIP2;7 at the plasma membrane during abiotic stress conditions. Thus, the autophagosome and MVB pathways act coordinately in downregulating the levels of aquaporin proteins at the plasma membrane to reduce water transport under abiotic stresses. Selective autophagy increases the degradation of newly synthesized aquaporin proteins at the ER and Golgi apparatus to reduce their transport to the plasma membrane. On the other hand, the MVBs pathway promotes degradation of those aquaporin proteins already localized in the plasma membrane through increased endocytosis. Through coordinated degradation of both newly synthesized aquaporin proteins at the ER and Golgi apparatus by autophagy and plasma membrane-localized aquaporin proteins by the endocytosis/MVB pathway, plant root cells can rapidly downregulate the water channel proteins at the cell surface to reduce root cell water permeability upon exposure to abiotic stresses (Figure 2).

*Arabidopsis* autophagy-deficient mutants are hypersensitive to heat stress, indicating an important role of autophagy in plant heat stress responses [18,81]. High temperature increases misfolded proteins and causes proteotoxic stress. Cellular responses to proteotoxic stress include chaperone-dependent refolding of misfolded proteins, degradation through the 26S proteasome system, and sequestration through aggregation [82]. Protein aggregates are potentially cytotoxic and are removed primarily by a specific type of selective autophagy termed aggrephagy [83]. In plants, misfolded protein aggregates are ubiquitinated by unknown E3 ligases and recognized by the selective autophagy receptor NBR1 through its ubiquitin-associated (UBA) domain [18,19,84]. NBR1 also interacts with ATG8, thereby tethering the protein aggregates to ATG8 for autophagic clearance in the vacuole. Arabidopsis *nbr1* mutants are hypersensitive to abiotic stresses, including high temperature, and this phenotype is associated with increased accumulation of ubiquitinated insoluble protein aggregates under heat stress [18,19,84]. These results indicate that NBR1-mediated aggrephagy plays a critical role in plant heat stress responses. There are additional pathways of selective autophagy that target misfolded proteins in different subcellular compartments under heat stress. In the ER, for example, an increase in misfolded proteins results in ER stress and triggers autophagy [15]. *Arabidopsis* ATG8-interacting ATI3 proteins also play a role in plant heat stress responses [85]. ATI3s interact with two closely related ER proteins UBAC2A (ubiquitin-associated protein 2a) and UBAC2B implicated in ER-associated protein degradation [85]. The *ati3* and *ubac2* mutants are compromised in both the heat tolerance and sensitivity to an ER stress-inducing agent [85]. These results support that ATI3 and UBAC2 participate in plant heat stress responses by targeting specific unknown ER components for autophagic degradation.

Heat stress increases misfolded proteins not only in intracellular compartments but also in the plasma membrane. Extensive studies in different eukaryotic systems have established that protein conformational surveillance and quality control of plasma-membrane proteins are necessary for cellular and organismal survival [86]. Like their intracellular counterparts, nonnative plasma membrane proteins are subjected to degradative quality control through four steps: (1) Recognition and ubiquitination, (2) endocytosis, (3) sorting into the intralumenal vesicles of MVBs, and (4) fusion of MVBs with the lysosomal/vacuolar compartment for degradation [86]. In plants, ubiquitination-mediated internalization and vacuolar clearance of plasma membrane proteins have also been demonstrated [87]. Furthermore, *Arabidopsis lip5* mutants are compromised in tolerance to heat stress and the heat sensitivity of the *lip5* mutant is associated with increased accumulation of ubiquitinated insoluble protein aggregates under heat stress [10]. Therefore, while autophagy plays a critical role in the clearance of nonnative protein aggregrates in the intracellular compartments, the endocytic and MVB pathway is critical in removal of nonnative plasma membrane proteins under heat stress (Figure 2). Ubiquitination of nonnative plasma membrane proteins in other eukaryotic organisms involves the conserved CHIP (C-terminus of Hsc70 interacting protein) E3 ligase [86]. Genetic analysis has shown that *Arabidopsis* mutants for the conserved CHIP E3 ligase are also compromised in heat tolerance and this phenotype is enhanced in the autophagy-deficient mutants [19]. The additive roles of CHIP- and autophagy-dependent protein degradation pathways further support the collective action of multiple protein degradation pathways under heat stress.

## 5. Autophagy and MVB Coordination in Plant Hormone-Mediated Regulation of Stress Responses

Plant hormones play important roles not only in plant growth and development but also in plant responses to biotic and abiotic stresses [19]. Among well-studied plant hormones, ABA (abscisic acid), ET (ethylene), JA (jasmonic acid), and SA (salicylic acid) are closely associated with plant stress responses, while others including auxin, CK (cytokinins), GA (gibberellic acid), and BRs (brassinosteroids) also participate in stress-triggered signaling [35]. Plant hormones can regulate autophagy and endocytic pathways in response to developmental and environmental cues. Autophagy genes, for example, can be regulated transcriptionally and posttranscriptionally by plant hormones, including ET, auxin, ABA, and SA [35]. Recent studies have also revealed that the critical roles of the endocytosis, MVB, and autophagy pathways in plant stress responses are in part mediated through their coordinated action in the metabolism, distribution, and signaling of important plant hormones, such as ABA, auxin, and BRs [35,88,89] (Figure 2).

ABA is an important plant hormone in seed maturation and germination and in plant response to both biotic and abiotic stresses. As discussed earlier, the multi-stress regulator TSPO is a selective autophagy receptor that mediates autophagic degradation of specific plasma membrane proteins, such as PIP2;7, aquaporin during plant stress responses [80]. TSPO is induced by ABA [90] and, thus, links the action of selective autophagy with ABA-regulated plant stress responses. Likewise, selective autophagy receptors ATI1 and 2 play a role not only in salt tolerance but also in seed maturation in response to ABA [91]. Therefore, the turnover of specific proteins through selective autophagy is a critical mechanism of ABA-regulated stress responses. The MVB pathway also plays a critical role in ABA-regulated stress responses through modulation of ABA signaling. ABA signaling involves a molecular module consisting of the PYR/PYL (pyrabactin resistance/pyrabactin resistance-like) ABA receptors, type 2C protein phosphatases (PP2Cs), and members of the sucrose non-fermenting-related kinase group 2 (SnRK2) family [89]. ABA binding of PYR/PYL ABA receptors promotes inhibition of PP2Cs [89]. When PP2Cs are inhibited, SnRK2s will remain phosphorylated and can activate ABA-responsive transcription factors [89]. PYR/PYL ABA receptors are soluble but are present at the membrane, most likely through interaction with a family of small proteins called CAR (C2-domain ABA-related) proteins that contain a lipid-binding C2 domain [92]. The E3 ligase RSL1 (ring finger of seed longevity 1), which contains a transmembrane domain and is localized to the plasma membrane and TGN, can ubiquitinate the PYR1 and PYL ABA receptors [93,94]. Furthermore, MVB ESCRT-I components FREE1 and VPS23A interact with PYR1/PYL4 and the levels of the ABA receptors increase in the *free1* and *vps23a* mutants [93,95]. These results indicate that ESCRT-I components FREE1 and VPS23a participate in ABA signaling by recognizing those PYR1/PYL4 ABA receptors ubiquitinated by the RSL1 E3 ligase and target their turnover by the MVB pathway. Very recently, it has been reported that FREE1 can also be phosphorylated by SnRK2 and relocate to the nucleus, where it interacts with ABA-responsive transcription factors and inhibits their DNA-binding activity [96]. Thus, both components from both autophagy and MVB pathways modulate the turnover and activity of ABA receptors and downstream regulators, including ABA-responsive transcription factors (Figure 2).

Auxin is a plant hormone with a cardinal role in the coordination of plant growth in response to developmental and environmental signals. One such auxin-regulated plastic plant body development in response to environmental stress is the formation of lateral roots under nutrient deficiency to increase nutrient acquisition from soil [97]. Lateral root development can also be inhibited as an avoidance strategy when plants are confronted with nutrient stress, high salinity, or heavy metals. Auxin is an important positive regulator of lateral root development and its directional transport through auxin efflux carriers, such as PIN (pin-formed), is critical [97]. Directional auxin transport promotes the establishment of auxin concentration gradients, and polar root cell growth during lateral root development is linked to the dynamic redistribution of PINs at the plasma membrane, which is tightly controlled by several protein trafficking pathways [98]. The plasma membrane-localized PINs can be internalized through clathrin-mediated endocytosis and can then be either recycled back to the plasma membrane or delivered to the vacuole for degradation through MVBs [98]. Using chemical biology, Perez-Henriquez and colleagues reported that the bioactive compound Sortin2 increases endosomal trafficking of plasma membrane recycling proteins, including PIN2, to the vacuole through MVBs and this action is important for Sortin2-induced lateral root initiation that is independent of the auxin receptor SCF^TIR^ [97]. These results support a pivotal role of endocytic trafficking of late endosome/MVB towards the vacuole in the induction of lateral roots. There are also reports on a role of autophagy in stress-induced lateral root formation. In *Arabidopsis*, lateral root formation under phosphate starvation requires the S-domain receptor kinase ARK2 and U box/Armadillo Repeat-containing E3 ligase PUB9 module [99,100]. The *ark2/pub9* mutant plants are defective in both lateral root formation and auxin accumulation in the root tips under phosphate starvation [99,100]. Interestingly, PUB9 is localized to autophagosomes upon phosphorylation by ARK2 or under phosphate starvation [99,100]. Inhibition of autophagic responses in *Arabidopsis* also leads to inhibition of both lateral root formation and auxin accumulation in the root tips [99,100]. These results indicate that autophagy is involved in the action of ARK2/PUB9 module in the regulation of lateral root development under phosphate starvation, likely through selective degradation of repressors of auxin accumulation and signaling (Figure 2).

Like auxin, BRs play important roles not only in plant growth and development but also in plant responses to environmental stresses, such as extreme temperatures and drought [88]. BRs are perceived at the cell surface by plasma membrane-localized receptors BR-INSENSITIVE-1 (BRI1), and its homologs, BRI1-LIKE-1 (BRL1) and BRL3. The binding triggers interaction with BRI1-ASSOCIATED-KINASE-1 (BAK1) (or members from the SOMATICEMBRYOGENESIS-RECEPTOR-KINASE (SERK) family) and the transphosphorylation of their kinase domains [88]. Activation of receptor complexes leads to a signaling cascade that relays BR signals to the accumulation of BRASSINAZOLERESISTANT-1 (BZR1) and BR-INSENSITIVE-EMS-SUPPRESSOR-1 (BES1) transcription factors in the nucleus, which control expression of BR-regulated genes [88]. Genetic analysis with BR-deficient or insensitive mutants have revealed BRs have a negative role in plant stress tolerance [101,102,103]. Both the endocytic and autophagy pathways can modulate components in BR signaling for downregulation of BR-regulated growth and upregulation of stress responses. First, the activity of BRI1 is under tight positive and negative regulation through phosphorylation, ubiquitination, endocytosis, and vacuolar degradation [104]. Upon BR perception, activated BRI1 phosphorylates PUB13 E3 ligase and stimulates their association and PUB13-dependent ubiquitination of BRI1 [104]. Ubiquitinated BRI1 undergoes clathrin-mediated endocytosis that is delivered through MVB trafficking to the vacuole for degradation [104]. Impaired internalization of BRI1 results in increased accumulation of BRI1 proteins in the plasma membrane and BR hypersensitivity [88]. Therefore, the MVB pathway plays a role in the attenuation of BR signaling (Figure 2). Second, autophagy also modulates BR responses through degradation of the BR-responsive transcription factors BES1 and BZR1 (Figure 2). Autophagy-dependent turnover of BEST1 is mediated by the autophagy receptor DSK2 (dominant suppressor of KAR2), which also interacts with ATG8 [102]. The interaction of DSK2 with ATG8 is activated upon phosphorylation of DSK2 by the GSK3-like kinase BIN2, a negative regulator in the BR pathway [102]. This mode of DSK2 regulation and turnover of BES1 integrates BR and autophagy pathways to achieve balances between growth and stress responses [88,102]. Similarly, carbon starvation leads to TOR inactivation, autophagy induction, and degradation of BZR1 transcription factor in the BR pathway [105]. By promoting the degradation of both BES1 and BZR1 transcription factors under stress conditions, autophagy helps modulate BR-promoted growth to promote stress responses. Intriguingly, studies using the application of exogenous BRs have indicated a positive role of BRs in plant tolerance to several stresses [106,107,108]. A recent study has reported that exogenous application of BR promotes autophagosome formation, decreases accumulation of ubiquitinated proteins, and enhances chlorophyll content under N starvation in tomato [109]. The protective role of exogenous BR in the plant response to N starvation is in part mediated by the action of the BZR1 transcription factor through upregulation of autophagy gene expression [109]. Therefore, the roles of BRs in plant stress responses are complex, which may reflect the dynamic nature of the activity of particular components in the BR and other associated signal transduction pathways that are subject to tight regulation by cellular processes, including autophagy and MVB trafficking.

## 6. Conclusions and Prospects

Studies in non-plant eukaryotic organisms, such as yeast, have established that autophagy and MVB pathways interact and coordinate at multiple levels in the regulation of cell growth and survival [28,29,30,31]. Recent studies have also made significant progress in revealing crosstalk and coordination of the two important trafficking pathways in plant cells [32,33]. In particular, a number of reported studies have identified components commonly associated with MVB and related trafficking pathways to be important in the biogenesis, maturation, and trafficking of autophagosomes in plant cells [32,33]. Both the autophagy and MVB pathways play critical roles in plant responses to a broad spectrum of biotic and abiotic stresses and, given the closely related nature of the two protein degradation pathways, it is highly expected that these two close protein degradation pathways are coordinated in the regulation and action in plant stress responses. In this review, we discussed recent progress in the analysis of a substantial number of components that are involved in the crosstalk and coordination between autophagy and MVB pathways in both their regulation and functions during plant responses to biotic and abiotic stresses (Figure 2, Table 1). Despite this progress, our understanding of the complex nature of the interactions between the autophagy and MVB pathways during the plant stress response is still very limited. In contrast to the large number of studies on stress-responsive autophagy, the role of the MVB pathway in the plant stress response has been examined only to a very limited extent, most likely due to la ack of appropriate mutants because of the essential role of MVBs in plant growth and development. With the identification of key regulators, such as LIP5, required for stress-induced MVB biogenesis, it is now possible to examine genetically the functional and mechanistic interactions between the autophagy and MVB pathways in the plant stress response through the generation and analysis of genetic mutants that are deficient in both stress-induced autophagosome and MVB biogenesis. Such studies will further our understanding of the important and dynamic roles of the cellular vesicle trafficking system in the complex network of plant stress responses.

## Figures and Tables

**Table 1:** Important *Arabidopsis* genes in autophagy and MVB (multivesicular body) coordination in plant stress responses.

Functional Group	Gene Name	AGI	Functional Description
Stress regulators	MAPK3	At3g45640	mitogen-activated protein kinases
	MAPK6	At2g43790	
	WRKY33	At2g38470	WRKY transcription factor
	TOR	At1g50030	Regulator of cell growth and autophagy
	SnRK1.1	At3g01090	SNF1-related protein kinases
	SnRK.1.2	At3g29160	
	SnRK2.2	At3g50500	
	SnRK2.3	At5g66880	
	SnRK2.6	At4g33950	
Components in autophagy and MVB and associated trafficking pathways	NBR1	At4g24690	Selective autophagy in aggrephagy
	TSPO	At2g47770	Multi-stress regulator
	ATI1	At2g45980	Selective autophagy receptors
	ATI2	At4g00355	
	ATI3A	At1g177880	Dicot-specific selective autophagy receptors
	ATI3B	At2g16575	
	ATI3C	At1g73130	
	DSK2	At2g17200	Ubiquitin/autophagy receptor
	UBAC2A	At3g56740	ATI3-interacting ER proteins implicated in ER stress responses
	UBAC2B	At2g41160	
	SH3P2	At4g34660	SH3 domain-containing protein functioning with ESCRT complexes
	FREE1	At1g20110	FYVE domain protein involved in NVB protein sorting
	VPS23a	At3g12400	Component of ESCRT-III complexes
	SKD1	At2g27600	Regulator of MVB biogenesis
	LIP5	At4g26750	Activator of SKD1
	Mon1	At2g28390	guanine-nucleotide exchange factor in maturation and fusion of late endosomes
	CCZ1	At1g80910	
	RabG3b	At1g22740	Members of GTP-binding protein Rab7 family
	RABG3f	At3g18820	
	CHMP1A	At1g73030	ESCRT-related proteins
	CHMP1B	At1g17730	
	ARK2	At1g65800	Receptor-like protein kinase
	RSL1	At2g26130	RING-type E3 ligase
	PUB9	At3g07360	Plant U-box E3 ligases
	PUB12	At2g28830	
	PUB13	At3g46510	
	CHIP	At3g07370	C-terminus of Hsc70-interacting E3 ligase
Proteins modulated by autophagy and MVB pathways	PIP2;1	At3g53420	Aquaporin proteins
	PIP2;7	At4g35100	
	PYR1/PYL1	At4g17870	ABA receptors
	PYR4/PYL4	At2g38310	
	PIN2	At5g57090	Auxin efflux carrier
	BRI1	At4g39400	Receptor-like protein kinase; BR receptor
	BES1	At1g19350	Transcription factors in BR signaling pathway
	BZR1	At1g75080	
